# Upfront surgery for stage IIIA/B non-small cell lung cancer: retrospective cohort study

**DOI:** 10.1093/bjsopen/zrae008

**Published:** 2024-03-21

**Authors:** Hongsheng Deng, Jun Liu, Xiuyu Cai, Shunjun Jiang, Weixiang Lu, Qing Ai, Jianfu Li, Shan Xiong, Xiangyun Qin, Wenhua Liang, Jianxing He

**Affiliations:** Department of Thoracic Surgery and Oncology, The First Affiliated Hospital of Guangzhou Medical University, State Key Laboratory of Respiratory Disease, National Clinical Research Centre for Respiratory Disease, Guangzhou Institute of Respiratory Health, Guangzhou, China; Department of Thoracic Surgery and Oncology, The First Affiliated Hospital of Guangzhou Medical University, State Key Laboratory of Respiratory Disease, National Clinical Research Centre for Respiratory Disease, Guangzhou Institute of Respiratory Health, Guangzhou, China; Department of General Internal Medicine, Sun Yat-sen University Cancer Centre, State Key Laboratory of Oncology in South China, Collaborative Innovation Centre for Cancer Medicine, Guangzhou, China; Department of Thoracic Surgery and Oncology, The First Affiliated Hospital of Guangzhou Medical University, State Key Laboratory of Respiratory Disease, National Clinical Research Centre for Respiratory Disease, Guangzhou Institute of Respiratory Health, Guangzhou, China; Department of Pharmacy, The First Affiliated Hospital of Guangzhou Medical University, State Key Laboratory of Respiratory Disease, National Clinical Research Centre for Respiratory Disease, Guangzhou Institute of Respiratory Health, Guangzhou, China; Department of Thoracic Surgery and Oncology, The First Affiliated Hospital of Guangzhou Medical University, State Key Laboratory of Respiratory Disease, National Clinical Research Centre for Respiratory Disease, Guangzhou Institute of Respiratory Health, Guangzhou, China; Department of Thoracic Surgery and Oncology, The First Affiliated Hospital of Guangzhou Medical University, State Key Laboratory of Respiratory Disease, National Clinical Research Centre for Respiratory Disease, Guangzhou Institute of Respiratory Health, Guangzhou, China; Department of Thoracic Surgery and Oncology, The First Affiliated Hospital of Guangzhou Medical University, State Key Laboratory of Respiratory Disease, National Clinical Research Centre for Respiratory Disease, Guangzhou Institute of Respiratory Health, Guangzhou, China; Department of Thoracic Surgery and Oncology, The First Affiliated Hospital of Guangzhou Medical University, State Key Laboratory of Respiratory Disease, National Clinical Research Centre for Respiratory Disease, Guangzhou Institute of Respiratory Health, Guangzhou, China; LinkDoc Technology Co. Ltd, Beijing, China; Department of Thoracic Surgery and Oncology, The First Affiliated Hospital of Guangzhou Medical University, State Key Laboratory of Respiratory Disease, National Clinical Research Centre for Respiratory Disease, Guangzhou Institute of Respiratory Health, Guangzhou, China; Department of Thoracic Surgery and Oncology, The First Affiliated Hospital of Guangzhou Medical University, State Key Laboratory of Respiratory Disease, National Clinical Research Centre for Respiratory Disease, Guangzhou Institute of Respiratory Health, Guangzhou, China

## Abstract

**Background:**

Stage III non-small cell lung cancer is a heterogeneous disease. Several international guidelines recommend neoadjuvant treatment before surgery; however, upfront surgery is the preferred approach for technically resectable non-small cell lung cancer in East Asia. The aim of this retrospective study was to evaluate the long-term outcomes of curative-intent upfront surgery in stage IIIA/B non-small cell lung cancer.

**Methods:**

Patients who underwent curative-intent upfront surgery with stage cIIIA/B non-small cell lung cancer were identified. The clinical and pathological variables and survival outcomes were evaluated.

**Results:**

Overall, 664 patients were identified, of whom 320 (48.8%) had N2 disease, 66.7% were males, 49.4% had a smoking history, and 61.2% had lung adenocarcinoma. Lobectomy was the most performed surgical procedure (84.9%). A total of 40 patients (6.02%) had positive margins (R1/R2). The grade III adverse event rate was 2.0% (13 of 664). The median follow-up was 30.6 (range 1.9–97.7) months. At follow-up, the mortality rate was 13.3% (88 of 664) and 37.2% of patients (247 of 664) had recurrence. Lung (101 of 247 (40.9%)) and brain (53 of 247 (21.5%)) were the most common sites of recurrence. The median overall survival was 60.0 (95% c.i. 51.5 to 67.6) months, with overall survival probability at 1, 2, 3, and 5 years being 89.6%, 77.8%, 67.2%, and 49.0% respectively. The R0 cohort showed an improved median overall survival compared with the R1/R2 cohort (67.4 *versus* 26.5 months respectively; *P* = greater than 0.001). The multivariable analysis revealed that age greater than or equal to 65 years (HR 1.51, 95% c.i. 1.08 to 2.12; reference = age less than 65 years), tumour size (greater than or equal to 5 cm (HR 2.13, 95% c.i. 1.41 to 3.21) and greater than or equal to 3 cm but less than 5 cm (HR 1.15, 95% c.i. 0.78 to 1.71); reference = less than 3 cm), and adjuvant treatment (chemotherapy (HR 0.69, 95% c.i. 0.49 to 0.96) and targeted therapy (HR 0.30, 95% c.i. 0.12 to 0.76); reference = none) significantly predicted overall survival.

**Conclusion:**

Upfront surgery is an option for the management of stage IIIA/B non-small cell lung cancer.

## Teaser

This study provides important real-world data, with a sufficient sample size, that upfront surgery for stage IIIA/B non-small cell lung cancer could have an acceptable perioperative morbidity rate and favourable long-term survival, particularly when combined with adjuvant therapies. This study identifies specific patient characteristics that may make them more suitable candidates for upfront surgery, such as younger age, smaller tumour size, limited nodal involvement, and no distant metastases. A comprehensive literature review on the survival rates of upfront surgery in non-small cell lung cancer facilitates easy comparison with relevant research findings in this field.

## Introduction

Stage III non-small cell lung cancer (NSCLC) is a heterogeneous disease^1^ and accounts for 22% of all cases of NSCLC^2,3^. Patients can present with disease that is potentially surgically resectable or with metastatic involvement^4,5^. Currently, most guidelines, including the National Comprehensive Cancer Network (NCCN)^6^, European Society for Medical Oncology (ESMO)^7^, American Society for Clinical Oncology (ASCO)^8^, and American College of Chest Physicians (ACCP)^9^ guidelines, recommend neoadjuvant therapy for stage IIIA-N2 NSCLC. ‘Upfront surgery’ refers to surgical resection as the initial treatment without prior radiation therapy or chemotherapy. This approach can provide radical cure and has been widely accepted as an optional first-line treatment for technically resectable stage III NSCLC in East Asia^10–12^, but not in all parts of the world.

There are no phase III RCTs directly comparing outcomes of upfront surgery followed by adjuvant chemotherapy *versus* induction therapy followed by surgery in stage III NSCLC, and the selection of the neoadjuvant therapy is based on extrapolation of phase I/II RCT data and expert consensus. The NATCH trial^13^ demonstrated identical disease survival rates for neoadjuvant *versus* adjuvant chemotherapy; however, it was conducted for stage IA (greater than 2 cm) to II NSCLC. Several studies^14–18^ failed to demonstrate the survival benefit of induction treatment when compared with upfront surgery. A study^17^ analysed the data of 1356 patients from the National Cancer Database (NCDB), demonstrating that postoperative chemotherapy showed comparable survival outcomes to preoperative chemotherapy in stage III/N2 NSCLC (HR 1.05; *P* = 0.438). Bertolaccini *et al*.^16^ concluded that upfront surgery, as the initial treatment for stage III/N2 NSCLC, yielded favourable recurrence-free survival (RFS), similar to induction chemotherapy followed by surgery (*P* = 0.93). Moreno *et al*.^18^ compared the impact of different treatment modalities in T3 (greater than 7 cm) N1 NSCLC and found that the 5-year overall survival (OS) rates were similar across neoadjuvant chemoradiation with surgery, neoadjuvant chemotherapy with surgery, surgery with adjuvant chemoradiation, and surgery with adjuvant chemotherapy modalities (40%, 44%, 40%, and 38% respectively). A meta-analysis by Lim *et al*.^15^ indicated that for stage IIIA-N2 NSCLC, the 5-year OS rate was 40.1% for the surgery-first approach followed by adjuvant chemotherapy and 39.3% for the chemotherapy-first approach followed by surgery. Surgical interventions after chemotherapy can be technically more challenging^19^ and neoadjuvant therapy might delay surgery and risk disease progression^11^.

Upfront surgery still remains a less commonly utilized approach for stage IIIA/B NSCLC, with definitive chemoradiotherapy (CRT) or CRT with durvalumab (PACIFIC pattern^20^) considered as the current standard of care. There is a lack of consensus regarding optimal candidate selection for upfront surgery and adjuvant treatment strategies for patients with stage IIIA-IIIB NSCLC, as well as insufficient research to reveal the long-term survival outcomes of upfront surgery in this patient cohort. The aim of this study was to assess the long-term outcomes of upfront surgery for patients with stage IIIA/B NSCLC in real-world practice, to identify prognostic factors that influence postoperative survival, and to discuss the selection criteria and definition of resectability for identifying optimal candidates who could benefit from upfront surgery.

## Methods

### Study design and patient inclusion/exclusion

This was a retrospective study of patients with stage cIIIA/B NSCLC (AJCC seventh or AJCC eighth) treated with upfront surgery between 1 January 2012 and 31 December 2019, from the structured electronic medical record (LinkDoc database) at The First Affiliated Hospital of Guangzhou Medical University. The inclusion criteria were as follows: patients who did not receive neoadjuvant therapy; patients greater than 18 years old; and patients who underwent surgical resection (lobectomy, bronchial sleeve resection, wedge resection, segmental resection, extended lobe resection, bi-lobectomy, or pneumonectomy). The exclusion criteria were as follows: patients enrolled in other clinical trials; patients with other malignancies at the time of diagnosis; and patients with missing data for treatment regimens.

Informed consent was waived due to the retrospective nature of the study. This study was approved by the ethics committee of The First Affiliated Hospital of Guangzhou Medical University (No. 2020-122).

### Upfront surgery strategy

All patients underwent a standard preoperative staging workup that included pretreatment tumour biopsy (through endobronchial ultrasound (EBUS) or percutaneous biopsy), chest CT, bronchoscopy, enhanced brain MRI or CT, bone scintigraphy, abdominal ultrasonography or CT, and various cardiopulmonary examinations. Additionally, PET/CT was selectively used for patients presenting with bulky mediastinal masses or with discrete lymph nodes that could not be distinguished or measured.

A multidisciplinary team consisting of a medical oncologist, a thoracic surgeon, and a radiologist was responsible for assessing resectability for patients with stage IIIA/B NSCLC. If there were no indications of extra-nodal tumour invasion, there was no evidence of direct tumour invasion into the great vessels, diaphragm, heart, trachea, and carina, and the involved lymph node was clearly distinguishable from surrounding tissues by CT/PET-CT, the tumour was considered resectable. Those patients with a tumour deemed resectable underwent lobectomy with systematic mediastinal lymph node dissection (greater than or equal to 6 lymph nodes and greater than or equal to 3 nodal stations^21^), with extension to anatomical pulmonary resection (such as bi-lobectomy or pneumonectomy) performed as needed. For patients with poor lung function, limited resection, such as wedge resection or segmentectomy with systemic lymphadenectomy, was deemed acceptable. According to prior research^22–24^ and following the GOLD report^25^, poor lung function was defined as post-bronchodilator forced expiratory volume in one second (FEV1)/forced vital capacity (FVC) less than 70%. Initial resection was conducted using video-assisted thoracoscopic surgery (VATS), with conversion to hybrid VATS or open surgery undertaken if necessary. All patients’ pathological stages were restaged according to the seventh edition^26^ of the Lung Cancer Stage Classification^27^. In our institution, surgery is rarely offered for patients diagnosed with N3 disease. Those scheduled for adjuvant therapy, without supraclavicular disease, with good cardiopulmonary performance, and with non-bulky nodal disease are candidates for surgery.

After upfront surgery, patients were assigned to one of several adjuvant treatment regimens based on driver mutation evaluation of postoperative pathological specimens, including chemotherapy, targeted therapy, CRT, anti-angiogenic therapy in combination with chemotherapy, or combined chemotherapy and targeted therapy. The administration of adjuvant therapy was not obligatory for patients who demonstrated intolerance to chemotherapy or other adjuvant treatments.

### Data collection and statistical analysis

Information regarding demographics, diagnosis, treatment, and surgical and oncological outcomes was extracted from the structured electronic medical record (LinkDoc database). The extracted data included: age, sex, smoking history, year of initial NSCLC diagnosis, histological subtype, pathological N status, clinical stage, year and type of resection, surgical margin, adjuvant therapy, date of recurrence, type of recurrence after resection, and date of death. OS was defined as the interval from the date of diagnosis to the date of death. RFS was taken as the time from the date of surgical resection until the first recurrence (locoregional or distant failure). Grade 3 or higher postoperative adverse effects were defined based on the Common Terminology Criteria for Adverse Events (CTCAE) version 3.0^28^.

The study estimated OS and RFS using the Kaplan–Meier method and stratified by resection margin (also proportionality assessed). For quantitative data, mean(s.d.) is used for normally distributed quantitative data and median (interquartile range (i.q.r.)) is used for skewed or non-normally distributed quantitative data. Cox proportional hazard models were used to identify potential prognostic factors for OS. A log rank test was used to compare survival between groups. Logistic regression was used to analyse relevant factors influencing complete resection. *P* < 0.050 was considered statistically significant.

## Results

### Patient characteristics

A total of 664 patients with stage cIIIA/B NSCLC met the eligibility criteria. The demographic and clinical characteristics of eligible patients are listed in *Table 1*. The majority of patients were male (65.7%), were younger than 65 years (70.5%), had lung adenocarcinoma (61.2%), and were cN2 (46.8%). Of these patients, 225 (33.9%) had a tumour size of less than 3 cm, 250 (37.7%) had a tumour size of greater than or equal to 3 cm but less than 5 cm, and 189 (28.4%) had a tumour size greater than or equal to 5 cm. A total of 118 patients (17.8%) underwent PET/CT for preoperative staging.

**Table 1:** Baseline characteristics and surgical/pathological outcomes of included stage III patients (*n* = 664)

Variable	Value
**Sex**	
Male	410 (65.71)
Female	214 (34.29)
**Age (years), mean(s.d.)**	58.8(9.94)
<65	440 (70.51)
≥65	184 (29.49)
**BMI (kg/m** ^2^ **), mean(s.d.)**	23.12(3.061)
**Race, Chinese, %**	100.0
Smoking history*	
Current/past	160 (29.04)/112 (20.33)
Never	279 (50.64)
**Histological subtype**	
LUAD	382 (61.22)
LUSQ	128 (20.51)
Other	114 (18.27)
**cT status**	
T1	225 (33.89)
T2	250 (37.65)
T3–T4	189 (28.46)
**Maximum tumour diameter (cm)**	
Mean(s.d.)	4.03(2.20)
Median (i.q.r.)	3.50 (2.50–5.00)
**cN status (based on preoperative staging)**	
N0	62 (9.34)
N1	269 (30.51)
N2	320 (48.19)
N3	13 (1.96)
**EGFR status†**	
Positive	110 (16.57)
Negative	158 (23.80)
**PD-L1 expression‡**	
<1%	91 (64.08)
1% to <25%	29 (20.42)
25% to <50%	7 (4.93)
≥50%	15 (10.56)

Values are *n* (%) unless otherwise indicated. *Smoking history data were unavailable for 48 patients. †EGFR status examination was not performed for 396 patients. ‡PD-L1 expression examination was not performed for 522 patients. LUAD, lung adenocarcinoma; LUSQ, lung squamous cell carcinoma; EGFR, epidermal growth factor receptor; PD-L1, programmed death ligand 1.

### Perioperative outcomes

See *Table 2*. A total of 624 patients (94.0%) had an R0 resection and positive margins were identified in 40 cases (6.0%), including 10 (1.5%) with microscopically positive (R1) margins and 30 (4.5%) with macroscopically positive (R2) margins. Lobectomy was performed in 84.9% of cases, sleeve lobectomy in 7.2% of cases, pneumonectomy in 4.5% of cases, wedge resection in 2.6% of cases, and segmentectomy in 0.8% of cases. The 30-day perioperative mortality rate was 1.36%, with a median duration of hospital stay of 17.0 (i.q.r. 14.0–22.0) days. Within the patient cohort, 13.10% experienced adverse events, with nausea (2.3%), anaemia (1.8%), and leucopenia (1.4%) being the most common. Other adverse events included hyperglycaemia (1.2%), polypnoea (0.9%), chest pain (0.9%), myelosuppression (0.6%), vomiting (0.6%), coughing (2.1%), and other events (1.4%). Grade III adverse events were reported in 2.0% of patients.

**Table 2:** Perioperative outcomes of all included patients (*n* = 664)

Variable	Value
**Surgical procedure**	
Pneumonectomy	30 (4.5)
Lobectomy	564 (84.9)
Sleeve lobectomy	48 (7.2)
Segmentectomy	5 (0.8)
Wedge resection	17 (2.6)
**Surgical margin**	
R0	624 (94.0)
R1	10 (1.5)
R2	30 (4.5)
**pN status (based on surgical specimens)**	
N0	51 (7.68)
N1	69 (10.39)
N2	521 (78.46)
Single-station metastasis	278 (53.35)
Multi-station metastasis	243 (46.64)
N3	23 (3.46)
**pTNM stage**	
IIIA	569 (91.19)
IIIB	55 (8.81)
IIIC	0
**Duration of perioperative hospital stay (days), median (i.q.r.)**	17.0 (14.0–22.0)
**Major postoperative complications**	
Intraoperative blood loss ≥500 ml	7 (1.1)
Prolonged air leak*	71 (10.7)
Postoperative pneumonia	46 (6.9)
Cardiac events	
Cardiac arrhythmia	9 (1.4)
Postoperative atrial fibrillation	2 (0.3)
Myocardial infarction	2 (0.3)
Wound infection	3 (0.5)
Acute respiratory failure	2 (0.3)
Pulmonary thromboembolism	3 (0.5)
Chylothorax	3 (0.5)
Bronchopleural fistula	1 (0.2)
Broncho-oesophageal fistula	1 (0.2)
Vocal cord palsy/recurrent laryngeal nerve paralysis	3 (0.5)
**Grade III adverse event**	13 (2.0)

Values are *n* (%) unless otherwise indicated. *A prolonged air leak was defined as a leak lasting more than 5 days. R0, R0 resection with negative margin; R1, R1 resection with microscopically positive margins; R2, R2 resection with macroscopically positive margins.

### Predictors for incomplete resection

Logistic regression was conducted to investigate the determinants of resection margins, comparing patients who underwent complete resection and incomplete resection. A total of four patients who underwent wedge resections due to palliative surgery were excluded from the analysis. When considering T and N stages separately, surgical procedure, T stage, N stage, and histological pathology were found to be associated with R1/R2 resection. Specifically, the OR for T4 compared with T1 was 21.46 (95% c.i. 1.74 to 265; *P* = 0.017), for N3 compared with N0 was 36.55 (95% c.i. 1.52 to 876; *P* = 0.026), for N2 compared with N0 was 5.06 (95% c.i. 0.72 to 35.7; *P* = 0.104), and for adenocarcinoma compared with squamous cell carcinoma was 0.21 (95% c.i. 0.055 to 0.829; *P* = 0.026). Sleeve resection, compared with lobectomy, had an OR of 10.53 (95% c.i. 2.49 to 44.49; *P* = 0.001) (*Table S1*). When considering TNM staging, stage IIIB, compared with stage IIIA, had an OR of 4.79 (95% c.i. 1.45 to 15.76; *P* = 0.010), indicating a stronger association with R1/R2 resection. Similarly, surgical procedure and histological type yielded consistent results, with an OR of 0.22 (95% c.i. 0.059 to 0.790; *P* = 0.020) for adenocarcinoma compared with squamous cell carcinoma and an OR of 6.05 (95% c.i. 1.60 to 22.85; *P* = 0.008) for wedge resection compared with lobectomy. However, BMI was found to be unrelated to incomplete resection (*Table S2*).

### Survival outcomes and recurrence pattern

Postoperative outcomes are shown in *Table 3*. After surgery, the following adjuvant therapies were received: 330 patients (49.70%) received chemotherapy; 65 patients (9.79%) received targeted therapy; 24 patients (3.61%) received chemotherapy combined with anti-angiogenic therapy; 23 patients (3.46%) received chemotherapy combined with radiotherapy; 8 patients (1.20%) received chemotherapy combined with targeted therapy; and 23 patients (3.46%) received other therapy.

**Table 3:** Postoperative outcomes of all included patients (*n* = 664)

Variable	Value
**Adjuvant therapy**	473 (71.23)
Chemotherapy	330 (49.70)
Targeted therapy	65 (9.79)
Chemotherapy + anti-angiogenic therapy	24 (3.61)
Chemotherapy + radiotherapy	23 (3.46)
Chemotherapy + targeted therapy	8 (1.20)
Other	23 (3.46)
Recurrence rate at the last follow-up time	247 (37.20)
**Recurrence site (for all patients with recurrence)**	
Lung	111 (50.45)
Brain	55 (25.00)
Skeleton	43 (19.54)
Liver	19 (8.64)
Lymph node	15 (6.82)
Other	41 (18.64)
**Recurrence-free survival probability, %**	
1.0 year	74.1
2.0 years	57.4
3.0 years	43.5
5.0 years	26.4
Overall survival (months), median (95% c.i.)	60.0 (51.5,67.6)
**Overall survival probability, %**	
1.0 year	89.6
2.0 years	77.8
3.0 years	67.2
5.0 years	49.0
**Survival status**	
Death	202 (30.42)
Live	372 (56.02)
Missing	90 (13.55)

Values are *n* (%) unless otherwise indicated.

**Fig. 1:**
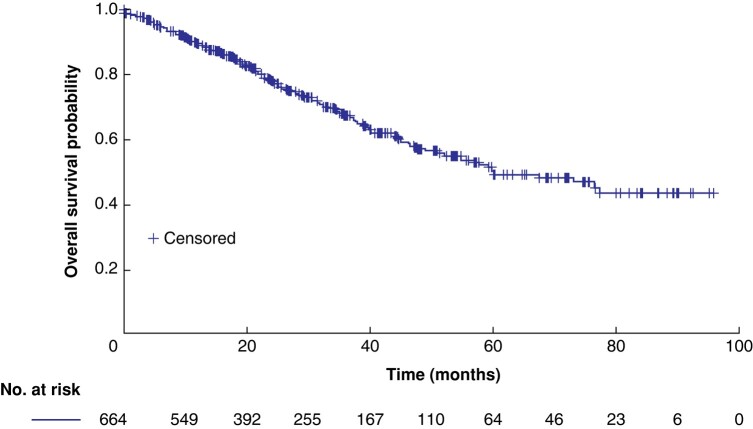
Kaplan–Meier curve of overall survival for the included patients

The median follow-up was 30.6 (range 1.9–97.7) months. At follow-up, the mortality rate was 13.3% (88 of 664) and 37.2% of patients (247 of 664) had evidence of disease recurrence. Recurrence was observed in 247 patients (37.20%); 101 of 247 patients (40.9%), 53 of 247 patients (21.5%), 45 of 247 patients (18.2%), and 19 of 247 patients (7.7%) developed lung, brain, skeleton, and liver metastasis respectively. The RFS probability at 1, 2, 3, and 5 years was 74.1%, 57.4%, 43.5%, and 26.4% respectively. Additionally, the median OS was 60.0 (95% c.i. 51.5 to 67.6) months (*Fig. 1*), with OS probability at 1, 2, 3, and 5 years being 89.6%, 77.8%, 67.2%, and 49.0% respectively. Survival status data revealed that 30.42% of patients had died at the time of analysis and that 56.02% of patients were alive at the time of analysis; data were missing for 13.55% of patients. R0 resection patients exhibited a median OS of 67.4 (95% c.i. 55.5 to 75.2) months, with 1-, 2-, 3-, and 5-year OS rates of 90.5%, 79.0%, 68.3%, and 50.2% respectively. In contrast, non-R0 resection patients had a median OS of 26.5 (95% c.i. 14.8 to 37.8) months, with 1-, 2-, and 3-year OS rates of 75.5%, 57.8%, and 48.9% respectively. Log rank test results indicated a significant disparity in OS between the two groups (*P* = greater than 0.001). Among R0 resection patients, the median disease-free survival was 30.8 (95% c.i. 25.7 to 34.7) months, with 1-, 2-, 3-, and 5-year disease-free survival rates of 74.7%, 58.2%, 44.4%, and 28.0% respectively. Meanwhile, non-R0 resection patients exhibited a median progression-free survival of 21.4 (95% c.i. 11.2 to 28.8) months, with 1-, 2-, and 3-year progression-free survival rates of 65.1%, 45.1%, and 30.1% respectively. However, the log rank test revealed no statistically significant difference in disease-free survival/progression-free survival between the two groups (*P* = 0.08).

### Independent prognostic factors after upfront surgery

Multivariable regression analysis was used to explore the factors affecting RFS and OS for patients who received upfront surgery, adjusted for age, sex, smoking status, histology, tumour size, surgical factors, pN/pT status, and adjuvant treatment (*Table 4*). The multivariable analysis revealed that age greater than or equal to 65 years (HR 1.51, 95% c.i. 1.08 to 2.12; reference = age less than 65 years), tumour size (greater than or equal to 5 cm (HR 2.13, 95% c.i. 1.41 to 3.21) and greater than or equal to 3 cm but less than 5 cm (HR 1.15, 95% c.i. 0.78 to 1.71); reference = less than 3 cm) and adjuvant treatment (chemotherapy (HR 0.69, 95% c.i. 0.49 to 0.96) and targeted therapy (HR 0.30, 95% c.i. 0.12 to 0.76); reference = none) significantly predicted OS.

**Table 4:** Multivariable Cox analyses for recurrence-free survival and overall survival of included patients

	Recurrence-free survival		Overall survival	
	HR (95% c.i.)	*P*	HR (95% c.i.)	*P*
**Age (reference = <65 years)**		0.1308		0.0174
≥65 years	1.230 (0.940,1.608)		1.508 (1.075,2.115)	
**Sex (reference = female)**		0.8596		0.6176
Male	0.970 (0.695,1.354)		1.120 (0.718,1.748)	
**Smoking status (reference = never a smoker)**		0.6545		0.9865
Past smoker	1.039 (0.717,1.505)		0.987 (0.621,1.569)	
Current smoker	1.156 (0.830,1.611)		0.966 (0.630,1.482)	
**Histology (reference = LUAD)**		0.0577		0.1625
LUSQ	0.736 (0.533,1.017)		1.156 (0.789,1.692)	
LASC	1.109 (0.525,2.345)		1.446 (0.600,3.486)	
Other	0.641 (0.434,0.948)		0.594 (0.337,1.046)	
**Tumour size (reference = <3 cm)**		0.0016		0.0003
≥3 cm but <5 cm	1.136 (0.855,1.510)		1.153 (0.778,1.707)	
≥5 cm	1.723 (1.258,2.358)		2.130 (1.413,3.209)	
**Surgical procedure (reference = pneumonectomy)**		0.4444	–	0.5678
Segmentectomy	0.274 (0.036,2.117)			
Sleeve lobectomy	0.738 (0.356,1.534)		1.108 (0.438,2.804)	
Wedge resection	1.186 (0.522,2.694)		2.247 (0.779,6.484)	
Lobectomy	0.950 (0.526,1.718)		1.277 (0.574,2.840)	
**Surgical margin (reference = R1/R2 resection)**		0.4907		0.2163
R0 resection	0.834 (0.499,1.396)		0.684 (0.375,1.249)	
**pN status (reference = pN0)**		0.2175		0.5473
pN1	0.854 (0.484,1.506)		1.203 (0.590,2.454)	
pN2	1.121 (0.706,1.777)		1.450 (0.778,2.706)	
pN3	1.820 (0.874,3.787)		1.832 (0.721,4.659)	
**Adjuvant treatment (reference = none)**		0.0835		0.0163
Chemotherapy	0.797 (0.612,1.040)		0.686 (0.492,0.959)	
Targeted therapy	0.512 (0.296,0.887)		0.297 (0.117,0.756)	
Other	0.854 (0.576,1.265)		0.599 (0.356,1.009)	

LUAD, lung adenocarcinoma; LUSQ, lung squamous cell carcinoma; LASC, lung adenosquamous carcinoma; R0, R0 resection with negative margin; R1, R1 resection with microscopically positive margins; R2, R2 resection with macroscopically positive margins.

## Discussion

This observational, retrospective study reports real-world data for long-term survival outcomes and identifies potential prognostic factors that influence the postoperative survival of stage IIIA/B NSCLC patients, treated with upfront curative-intent surgery at a single thoracic oncology centre. In the present study, with a median follow-up of 30.6 months, the median OS was(95% c.i. 51.5 to 67.6) months, with OS probability at 1.0, 2.0, 3.0, and 5.0 years being 89.6%, 77.8%, 67.2%, and 49.0% respectively. These results exceed the median OS of 52.5 (95% c.i. 43.1 to 61.9) months reported by Jung *et al*.^12^ based on a Korean cohort of stage III surgically resected NSCLC and are comparable to the median OS of 63 months observed in a German cohort of patients with radically resected NSCLC, including those with stage IIIB disease^29^. The present study’s 5-year OS rate is consistent with those reported for large real-world cohorts of stage cN2/pN2 NSCLC patients who underwent upfront surgery during a similar interval, with 5-year OS rates ranging from 43%^10,30^ to 44.7%^11,31^. The improved survival may be attributed to the higher number of patients aged younger than 65 years and a greater number of patients who received adjuvant treatments, such as targeted therapy or chemotherapy. The literature comparing the outcomes of upfront surgery for stage III NSCLC is summarized in *Table 5*, highlighting that studies (with similar research intervals) with high adjuvant treatment rates have favourable 5-year OS rates (43–71%^10,11,30,31,40,42^), 5-year RFS rates (21–50.6%^10,11,39,42^), and median OS (44.5–57.8 months^10,11,42,44^).

**Table 5:** World literature comparing the outcomes of upfront surgery for stage III NSCLC

Reference (publication year)	Adjuvant treatment rate, %	Tumour stage	Number of patients	Outcome	Months (median)	Five-year survival rate, %
Roth *et al*.^32^ (1998)	0	cIIIA	21	OS	7.0	0
Rosell *et al*.^33^ (1999)	S + R 100	cIIIA	30	OS	10.0	0
Andre *et al*.^34^ (2000)	S + R 65	cN2/pN2	562	OS	pN2L2 16; cN2 14	mN2L1 34; mN2L2 11; cN2L1 8; cN2L2 3*
Ichinose *et al*.^30^ (2001)	S + C 41; S + R 15	pIIIA-N2	402	OS	–	Total 31; pN2L1 43; pN2L2 17
Nagai *et al*.^35^ (2003)	0	cIIIA	31	OS	Total 16.0	Total 22
Casali *et al*.^36^ (2005)	S + C 100	pN2	183	OS	Total 24.0; pN2L1 25.2; pN2L2 16.0	Total 20; pN2L1 23.8; pN2L2 14.7
Ratto *et al*.^37^ (2009)	S + C 5.4; S + R 39.4; S + CR 25.6	cN2	192	OS	18.7	–
Hishida *et al*.^38^ (2014)	S + C 6.67	pIIIA-N2	45	RFS	–	22.3
				OS	–	23.6
Hancock *et al*.^39^ (2015)	S + C 22.4; S + R 12.5; S + CR 35.1	pIII NSCLC with R1 resection	177	OS	–	No adjuvant 12; S + C 18; S + R 9; S + CR 30
Maniwa *et al*.^40^ (2016)	45.8	cN2	29	OS	–	71.1
				DFS	–	50.6
Zheng *et al*.^10^ (2016)	S + C 84.6; S + R 23.5	pIIIA	668	PFS	17.1	21
				OS	44.5	43
Maniwa *et al*.^41^ (2018)	S + C 47.8; S + T 21.3	cN2	94	OS	Total 58.0; cN2pN2 48.0	Total 47.9; cN2pN0/1 74.9; cN2pN2 41.2
Yuan *et al*.^42^ (2019)	S + C 95.9; S + CR 33.3	pIIIA-N2	363	RFS	–	S + C 23.1; S + CR 15.5
				OS	S + C 50.0; S + CR 50.0	S + C 47.0; S + CR 44.5
Yun *et al*.^11^ (2019)	Total 82.6; S + C 23.9; S + R 17.4; S + CR 35.1	pN2	706	RFS	23	33.8
				OS	52	44.7
Taber and Pfannschmidt^43^ (2020)	Total 29.3; S + C 17.9; S + R 2.9; S + CR 8.5	cIIIA	250	DFS	–	24.9
				OS	–	30.0
		cIIIB	94	DFS	–	8.3
				OS	–	8.3
Jazieh *et al*.^44^ (2021)	100	IIIA	114	PFS	18.8	–
				OS	57.8	–
		IIIB	13	PFS	13.8	–
				OS	34.7	–
Bertolaccini *et al*.^16^ (2022)	S ± C NR	pN2	126	OS	R0 66.0	R0 66.3
				RFS	–	R0 22
Bitenc *et al*.^31^ (2022)	S ± C/R NR	cIIIA/pIIIA	85	OS	cIIIA not reached; pIIIA 51.6	cIIIA 55.3; pIIIA 49.9
Hayakawa *et al*.^45^ (2023)	S ± C NR	cN2pN2	43	RFS	–	No adjuvant 28.0; S+ C 56.5
				OS	–	No adjuvant 41.9; S + C 82.2
Fu *et al*.^46^ (2023)	S + C 85.7; S + R 23.4	pIIIA-N2	475	OS	R0 48.1; R1/2 28.0; pN2L1 61.0; pN2L2 35.0	42.2
				RFS	R0 18.0; R1/2 10.6; pN2L1 21.7; pN2L2 12.0	21.3
Campisi *et al*.^47^ (2023)	S + C 71.2; S + CR 21.2	cN2pN2	52	OS	37.00	30.8
				RFS	27.96	–
The present study (2024)	Total 71.23; S + C 49.70; S + T 9.79	cIIIA/IIIB	664	RFS	R0 30.8; R1/2 21.4	R0 28.0; R1/2 0
				OS	Total 60.0; R0 67.4; R1/2 26.5	Total 49.0; R0 50.2; R1/2 0

*Patients with minimal N2 (mN2) are those with no preoperative detection of N2 disease, and patients with cN2 are those in whom N2 disease is detected before surgery by CT and confirmed by mediastinoscopy. Multiple lymph node levels involved is defined as L2, and a single lymph node level involved is defined as L1. OS, overall survival; S, surgery; R, adjuvant radiotherapy; C, adjuvant chemotherapy; CR, complete response; RFS, recurrence-free survival; NSCLC, non-small cell lung cancer; R1, R1 resection with microscopically positive margins; DFS, disease-free survival; PFS, progression-free survival; T, adjuvant targeted therapy; NR, not reported; R0, R0 resection with negative margin; R2, R2 resection with macroscopically positive margins.

The main challenge regarding upfront surgery for stage III NSCLC is selecting suitable patients and optimizing the adjuvant strategy. The involvement of lymph nodes, as determined by imaging or intraoperative examination^48^, can impact the extent and feasibility of surgical intervention and may require modification of the planned surgical procedure. Whereas some centres consider ipsilateral non-bulky single nodal station involvement or discrete non-diffuse mediastinal nodes as a surgical indication^49^, others advocate a more aggressive surgical intervention for NSCLC with mediastinal nodes over 10 mm^50^. According to Bertolaccini *et al*.^16^, upfront surgery may be appropriate for individuals with single-station mediastinal nodal involvement and no evidence of extra-nodal tumour invasion. Maniwa *et al*.^40^ specified four criteria for performing upfront surgery for patients with cN2 NSCLC (single-station N2 disease, non-bulky N2 disease, N2 disease with regional mode of spread, and N2 disease without N1 disease) and these patients had a 5-year OS rate of 71.1%. Hishida *et al*.^38^ also stated that true single-station pathological N2 and negative subcarinal node status were independent prognostic factors that favoured survival after initial resection for clinical single-station N2/pathological N2 patients, with an HR of 0.35 (*P* = 0.008) and 0.34 (*P* = 0.022) respectively. Bulky multi-station N2 disease is frequently not amenable to surgery. In the present study, approximately 51.71% of patients who underwent upfront surgery had multi-station N2 metastasis; this multi-station rate is consistent with previous literature^30,42^ and indicates that surgery is still feasible in these patients.

Although it is technically feasible to remove large and locally invasive tumours, there is limited evidence supporting extended resections for stage N3 NSCLC^51^. In the present study, 13 patients (1.96%) with N3 disease received upfront surgery. The best approach for stage N3 NSCLC is still uncertain, especially for patients who are fit for surgery. It is plausible that these patients could be treated similarly to those with operable stage IIIA (N2) NSCLC and achieve satisfactory outcomes. A previous study^52^ has demonstrated that, for patients with cN3 or pN3 stage NSCLC, surgery was associated with improved long-term survival compared with chemoradiation (HR 0.76, 95% c.i. 0.58 to 0.99). Similarly, an analysis of the Surveillance, Epidemiology, and End Results (SEER) database demonstrated that patients with N3 stage NSCLC who received surgery had significantly better survival (HR 0.71, 95% c.i. 0.64 to 0.79; *P* < 0.001) compared with those who did not^53^. These studies suggest that, in carefully selected patients with limited N3 disease, surgery may offer superior survival and can be considered as part of a multimodal therapeutic approach.

Although surgical resection is the mainstay treatment for NSCLC, positive margins occur in between 10%^54^ and 12.6%^55^ and are associated with worse long-term outcomes, essentially decreasing the 5-year OS by half^39,56–58^. Riquet *et al*.^58^ reported a median OS of 17 months for R1 resection and 51 months for R0 resection. R0 resection is crucial for upfront surgery. In the present study, the incidence of R1/R2 resection was 6.02%, which is consistent with previous literature^39,58^, and was significantly associated with worse survival compared with complete resection (median OS of 26.5 *versus* 67.4 months; *P* < 0.001). In addition, stage IIIB (particularly involving stage T4 and N3), lung squamous cell carcinoma, and sleeve resection all exhibited a heightened incidence of positive margins, aligning with findings from prior studies^58,59^. The identification of these factors that associated with incomplete resection may help guide clinical decision-making for upfront surgery and improve surgical outcomes.

While upfront surgery alone has demonstrated unfavourable 5-year OS rates, typically 14–30%^50,60–62^ for stage III NSCLC (*Table 5*), the addition of adjuvant treatment has been shown to lead to further improvement in survival. Examples include studies reporting enhanced median OS and 5-year rates (around 43.0%^10^ to 44.7%^11^) in stage cN2 and pIIIA NSCLC patients who received adjuvant treatments. Trials such as EVIDENCE^63^, ADAURA^64^, and IMpower010^65^ explored the effectiveness of adjuvant icotinib, osimertinib, and atezolizumab respectively, showing significant improvements in disease-free survival compared with chemotherapy. The encouraging results from trials involving adjuvant immunotherapy^65,66^ and targeted therapy^63,64^, surpassing adjuvant chemotherapy, suggest a potential shift toward a more efficacious treatment strategy involving upfront surgery followed by adjuvant therapy in the future.

Currently, most guidelines^6–8^ recommend induction therapy for stage IIIA-N2 NSCLC. For example, the ASCO guidelines^8^ recommend that patients with stage III N2 NSCLC scheduled to undergo surgical resection should receive neoadjuvant chemotherapy or neoadjuvant concurrent chemoradiation. The authors’ team^67^ has also found that neoadjuvant chemotherapy followed by surgery and adjuvant radiotherapy outperformed surgery alone and surgery followed by adjuvant radiotherapy through a network meta-analysis. However, not all patients can benefit from neoadjuvant treatment, with the pCR rate ranging from 0%^68^ to 53%^69^, depending on the specific regimen (chemotherapy/CRT^70^, targeted therapy^68^, or immunotherapy/immunochemotherapy^71,72^), and the persistent N2^73^ rate ranging from approximately 39.4%^74^ to 53%^73,75^. This suggests that nearly half of patients do not respond positively or, at the very least, do not experience a complete response to neoadjuvant treatment. The benefits observed in this subset of patients may amplify the overall benefits for those who undergo neoadjuvant chemotherapy, as substantiated by the provided references. Given that persistent N2 implies minimal benefit from induction therapy^76^ (in stage cN2 NSCLC patients, those who exhibit persistent ypN2 after neoadjuvant therapy have comparable postoperative survival to those who undergo upfront surgery^34^), it is logical to consider the possibility of shifting surgery before chemotherapy for these patients. To select suitable candidates for upfront surgery, in addition to meeting the general surgical indications^8^ (such as achieving complete resection (R0) of both the primary tumour and the involved lymph nodes, N3 lymph nodes not involved, and an expected low perioperative mortality), the utilization of predictive efficacy biomarkers can also be instrumental in determining the need for neoadjuvant therapy^77^.

This supports the principles of personalized medicine, aiming to identify individuals within the stage III NSCLC patient population who may not directly benefit from neoadjuvant treatment. Moving surgery upfront under such circumstances could be justifiable to avoid surgery delays that could result in disease progression^78^ or lead to the experience of side effects^79^ that might preclude surgery (*Fig. S1*). Similarly, the international guidelines for the management of malignant pleural mesothelioma (MPM)^80^ state that chemotherapy has limited efficacy and surgery may be appropriate for carefully and highly selected patients. Although these guidelines predominantly address MPM, the perception of the surgical role remains consistent, highlighting surgery as a viable option when deemed suitable, particularly in situations where systemic therapy may offer limited efficacy.

Overall, the present study indicates that, for patients with stage IIIA/IIIB NSCLC, upfront surgery can serve as an alternative therapeutic approach, provided that a comprehensive evaluation of technical resectability is performed and that adjuvant therapy is administered. Several limitations should be acknowledged. First, selection bias is inherent in a retrospective study from a single institution. Secondly, the heterogeneity of the surgical procedure might bias the results. Thirdly, clinical staging of the mediastinum is challenging and can be inaccurate.

## Supplementary Material

zrae008_Supplementary_Data

## Data Availability

Data are available upon reasonable request.
