# Supplementary material for: Upfront surgery for stage IIIA/B non-small cell lung cancer: retrospective cohort study
Source: BJS Open. 2024 Mar 21;8(2):zrae008. doi: 10.1093/bjsopen/zrae008 (PMC10957167; doi:10.1093/bjsopen/zrae008)

**Title**

**Upfront surgery for Stage IIIA-B Non-small cell lung cancer: Retrospective Cohort Study**

Hongsheng Deng^1#^MD, Jun Liu^1#^MD, Xiuyu Cai^2#^MD, Shunjun Jiang^1 3#^MD, Hengrui Liang^1^MD, Bo Cheng^1^MD, Weixiang Lu^1^MD, Qing Ai^1^MD, Jianfu Li^1^MD, Shan Xiong^1^MD, Xiangyun Qin^4^MD, Wenhua Liang^1*^MD, Jianxing He^1*^MD

^1^ Department of Thoracic Surgery and Oncology, the First Affiliated Hospital of Guangzhou Medical University, State Key Laboratory of Respiratory Disease, National Clinical Research Center for Respiratory Disease, Guangzhou Institute of Respiratory Health, Guangzhou, China

^2^ Department of General Internal Medicine, Sun Yat-sen University Cancer Centre, State Key Laboratory of Oncology in South China, Collaborative Innovation Centre for Cancer Medicine, Guangzhou 510060, China

^3^ Department of Pharmacy, the First Affiliated Hospital of Guangzhou Medical University, State Key Laboratory of Respiratory Disease, National Clinical Research Center for Respiratory Disease, Guangzhou Institute of Respiratory Health, Guangzhou, China.

^4^ LinkDoc Technology Co. Ltd, Beijing, China

**Corresponding author.**

Wenhua Liang and Jianxing He,

No.151, Yanjiang Road, Guangzhou 510120, China.

E-mail addresses: [liangwh1987@163.com](mailto:liangwh1987@163.com); [drjianxing.he@gmail.com](mailto:drjianxing.he@gmail.com)

Tel:+86-20-83337792;

ORCID ID: ; [0000-0003-4268-3324](https://orcid.org/0000-0003-4268-3324)

**Supplementary Materials - Index**

| **Supplementary Figures and Tables** |  |
| --- | --- |
| Supplementary Table 1 | *page 2* |
| Supplementary Table 2 | *page 3* |

Supplementary Table 1: Multivariate logistic regression analysis for predicting incomplete resection (With TNM stage alone a factor).

| Variable | B | Standard Error | Wald | df | p-value | OR | 95% CI (Lower) | 95% CI (Upper) |
| --- | --- | --- | --- | --- | --- | --- | --- | --- |
| Surgical Procedure (Reference: Lobectomy) | | | | | | | | |
| Sleeve Resection | 1.800 | 0.678 | 7.046 | 1 | 0.008 | 6.050 | 1.602 | 22.853 |
| Pneumonectomy | 0.456 | 1.118 | 0.166 | 1 | 0.684 | 1.577 | 0.176 | 14.121 |
| Histological Type (Reference: Squamous Cell Carcinoma) | | | | | | | | |
| Adenocarcinoma | -1.530 | 0.660 | 5.371 | 1 | 0.020 | 0.217 | 0.059 | 0.790 |
| Other | -1.787 | 1.102 | 2.629 | 1 | 0.105 | 0.168 | 0.019 | 1.452 |
| BMI (Reference: >28) | | | | | | | | |
| 24-28 | -17.961 | 6942.064 | 0.000 | 1 | 0.998 | 0.000 | 0.000 | . |
| 18.5-24 | -0.120 | 1.100 | 0.012 | 1 | 0.913 | 0.887 | 0.103 | 7.666 |
| <18.5 | -0.930 | 1.229 | 0.573 | 1 | 0.449 | 0.395 | 0.036 | 4.384 |
| Stage (Reference: Stage IIIA) | | | | | | | | |
| Stage IIIB | 1.566 | 0.608 | 6.630 | 1 | 0.010 | 4.786 | 1.453 | 15.761 |

(B), standard errors, Wald statistics, degrees of freedom (df), p-values, odds ratios (OR) and 95% confidence intervals for the odds ratios.

Supplementary Table 2: Multivariate logistic regression analysis for predicting incomplete resection (With T and N stage included separately).

| Variable | B | Standard Error | Wald | df | p-value | Exp(B) | 95% CI (Lower) | 95% CI (Upper) |
| --- | --- | --- | --- | --- | --- | --- | --- | --- |
| Surgical Procedure (Reference: Lobectomy) | | | | | | | | |
| Sleeve Resection | 2.355 | 0.735 | 10.263 | 1 | 0.001 | 10.534 | 2.494 | 44.488 |
| Pneumonectomy | 0.419 | 1.143 | 0.134 | 1 | 0.714 | 1.521 | .162 | 14.298 |
| Histological Type (Reference: Squamous Cell Carcinoma) | | | | | | | | |
| Adenocarcinoma | -1.545 | 0.692 | 4.981 | 1 | 0.026 | 0.213 | 0.055 | 0.829 |
| Other | -1.550 | 1.177 | 1.735 | 1 | 0.188 | 0.212 | 0.021 | 2.130 |
| BMI (Reference: >28) | | | | | | | | |
| 24-28 | -18.190 | 6640.192 | 0.000 | 1 | 0.998 | 0.000 | 0.000 | - |
| 18.5-24 | -0.118 | 1.178 | 0.010 | 1 | 0.920 | 0.889 | 0.088 | 8.942 |
| <18.5 | -0.975 | 1.312 | 0.552 | 1 | 0.457 | 0.377 | 0.029 | 4.933 |
| T Stage (Reference: T1) | | | | | | | | |
| T2 | 0.454 | 1.167 | 0.152 | 1 | 0.697 | 1.575 | 0.160 | 15.499 |
| T3 | 1.107 | 1.251 | 0.783 | 1 | 0.376 | 3.025 | 0.261 | 35.095 |
| T4 | 3.066 | 1.283 | 5.715 | 1 | 0.017 | 21.461 | 1.737 | 265.105 |
| N Stage (Reference: N0) | | | | | | | | |
| N1 | -0.878 | 1.325 | 0.439 | 1 | 0.508 | 0.416 | 0.031 | 5.580 |
| N2 | 1.621 | 0.997 | 2.642 | 1 | 0.104 | 5.057 | 0.716 | 35.706 |
| N3 | 3.599 | 1.621 | 4.926 | 1 | 0.026 | 36.546 | 1.523 | 876.838 |

(B), standard errors, Wald statistics, degrees of freedom (df), p-values, odds ratios (Exp(B)), and 95% confidence intervals for the odds ratios.

Supplementary Figure 1. Proposed criteria for identifying candidates for upfront surgery involve the evaluation of neoadjuvant efficacy biomarkers to prevent treatment delays and minimize adverse effects.


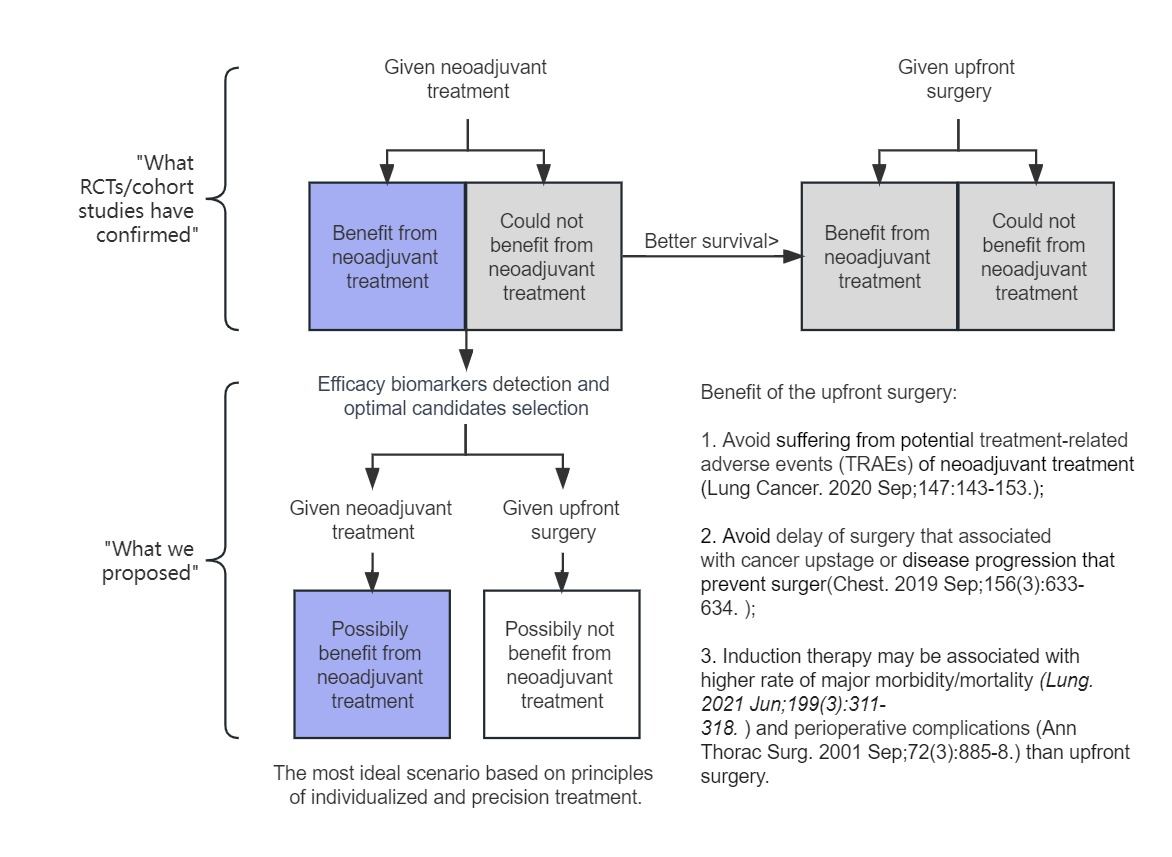

Supplement: zrae008_Supplementary_Data [file zrae008_supplementary_data.docx]
